# Waveguide-integrated spatial mode filters with PtSe_2_ nanoribbons

**DOI:** 10.1515/nanoph-2024-0552

**Published:** 2025-01-23

**Authors:** Tianping Xu, Zhengkun Xing, Shuqi Xiao, Rui Niu, Quan Yuan, Luping Xu, Zunyue Zhang, Tiegen Liu, Hon Ki Tsang, Jiaqi Wang, Zhenzhou Cheng

**Affiliations:** School of Precision Instrument and Opto-electronics Engineering, 12605 Tianjin University, Tianjin, China; Key Laboratory of Opto-electronic Information Technology, Ministry of Education, Tianjin, China; Department of Electronic Engineering, The Chinese University of Hong Kong, Hong Kong, China; College of Physics and Optoelectronic Engineering, Shenzhen University, Shenzhen, China; 12605 Georgia Tech-Shenzhen Institute, Tianjin University, Shenzhen, China; School of Physics and Electronic Engineering, Xinjiang Normal University, Urumqi, China

**Keywords:** silicon photonics, low-dimensional material, mode-division multiplexing

## Abstract

Low-dimensional material-based heterogeneous silicon photonics has attracted significant attention due to their applications in developing integrated optoelectronic devices from the telecommunication band to mid-infrared wavelengths. However, the study of waveguide components integrated with low-dimensional materials for mode-division multiplexing (MDM) applications mostly remains in its infancy. In this paper, we demonstrated waveguide-integrated spatial mode filters by integrating subtly designed ten-layer PtSe_2_ nanoribbons on an ultrathin silicon waveguide with a deep-subwavelength thickness to eliminate modal crosstalk. To be specific, the undesirable propagating mode can be filtered out due to its strong interaction with the PtSe_2_ nanoribbons on the silicon waveguide surface. Our results show that TE_1_-to-TE_0_ and TE_2_-to-TE_0_ modal extinction ratios of 12 dB and 14.5 dB were measured in 100 and 75-μm-long PtSe_2_-on-silicon waveguides at 2200-nm wavelengths. Our study paves the intriguing approach to developing waveguide-integrated spatial mode filters for on-chip MDM applications for optical interconnects and optical communications.

## 1 Introduction

Mode-division multiplexing (MDM) techniques have been extensively explored to meet the rapidly growing demands of communication bandwidth [1], [2]. Different from wavelength-division multiplexing techniques [3], MDM techniques increase data transmission density by utilizing monochromatic lasers [4], [5], which do not suffer from high costs, thermal management complexity, and large system footprints. It, therefore, attracts significant attention for MDM applications based on photonic integrated circuits (PICs) [6], [7]. Moreover, silicon photonics provides a promising platform for developing on-chip optical interconnects and optical communications combined with MDM techniques [8] with the advantages of negligible optical loss at 2–2.5 μm wavelengths and intrinsic compatibility with the complementary metal oxide semiconductor (CMOS) technology for high-volume and cost-efficient device fabrication [9], [10], [11], [12], [13], [14]. Nowadays, diverse studies of on-chip MDM silicon waveguide devices have been proposed and demonstrated, namely, spatial mode multiplexers/demultiplexers [15], bend waveguides [16], and waveguide crossings [17], [18].

On the other hand, low-dimensional material-based heterogeneous silicon photonics has been widely studied in the past decade, ranging from the telecommunication band to mid-infrared wavelengths [19], [20], [21]. Compared with bulk semiconductors, low-dimensional materials have the merits of tailorable electronic bandgaps, neglectable lattice mismatch with silicon-on-insulator (SOI) wafers, and excellent stability. To date, many great efforts have been made to demonstrate waveguide-integrated light sources [22], modulators [23], [24], [25], and photodetectors [26], [27], [28] based on graphene [29], [30], [31], [32], black phosphorus [33], [34], and transition metal dichalcogenides [35], [36], [37]. However, the study of waveguide components integrated with low-dimensional materials for MDM applications has seldom been experimentally explored.

In this paper, we demonstrated waveguide-integrated spatial mode filters with PtSe_2_ nanoribbons to eliminate modal crosstalk at 2-μm wavelengths for on-chip MDM applications. Due to the strong interaction between light in an ultrathin silicon waveguide with a deep-subwavelength thickness and subtly designed PtSe_2_ nanoribbons, optical modes with different orders can be intensely and selectively absorbed. To be specific, we experimentally achieved a TE_1_-to-TE_0_ modal extinction ratio (ER) of 12 dB and a TE_2_-to-TE_0_ modal ER of 14.5 dB in PtSe_2_-on-silicon waveguides at 2200-nm wavelengths. Our study offers a new approach to developing on-chip spatial mode filters for MDM applications.

## 2 Device structure and theoretical analysis

**Figure 1:**
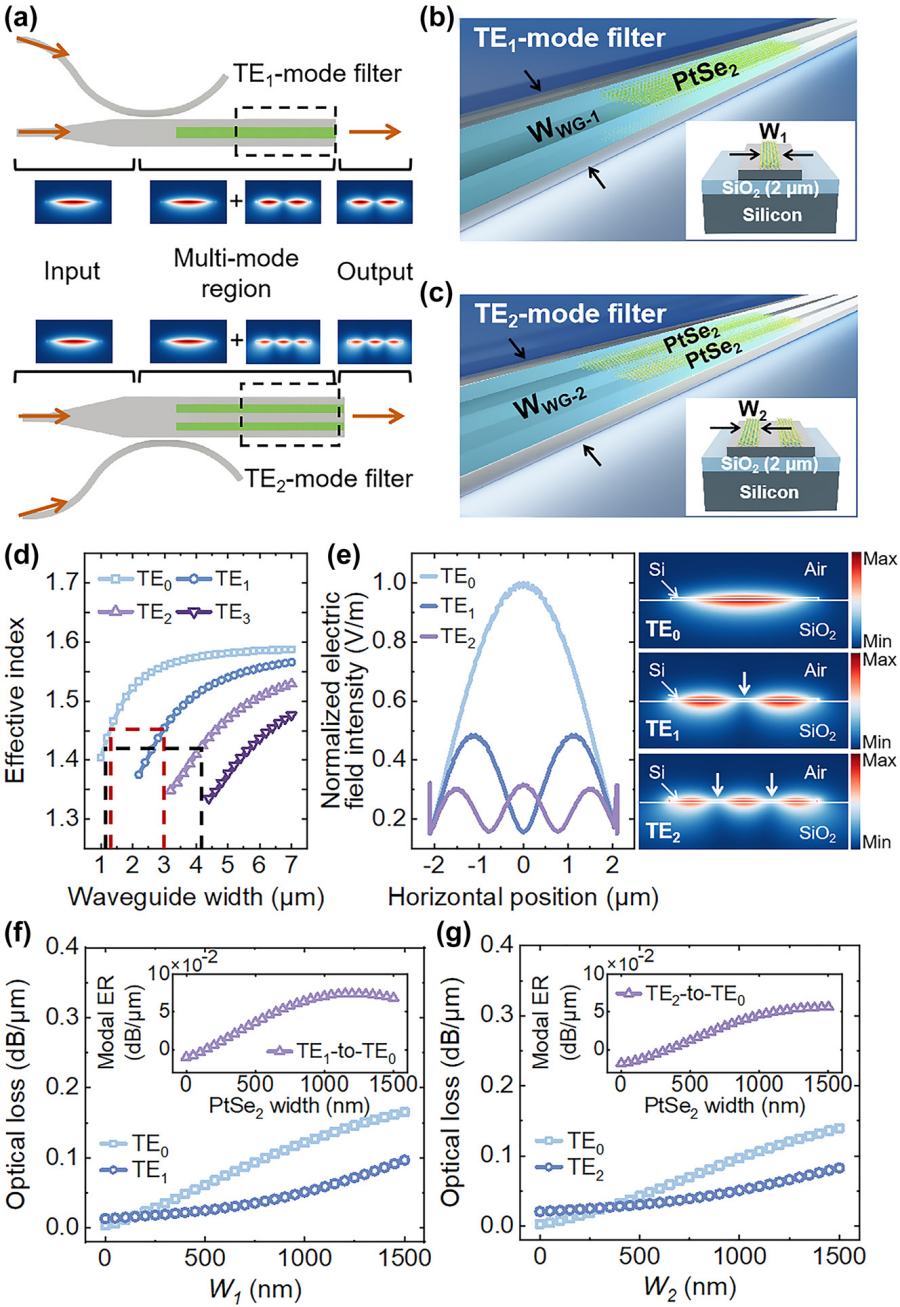
Schematics and theoretical results of the spatial mode filters. (a) Schematics of the devices, including the input single-mode waveguides, asymmetric directional couplers, and multi-mode waveguides integrated with the PtSe_2_ nanoribbons. 3D and cross-section views of the (b) TE_1_-mode filter and (c) TE_2_-mode filter. (d) Simulated effective RIs of the eigenmodes in the ultrathin silicon waveguides with different widths. (e) Normalized electric-field intensity profiles across the pure silicon waveguide and simulated electric-field distributions of the TE_0_, TE_1_, and TE_2_ modes. Arrow symbols indicate the positions of the PtSe_2_ nanoribbons in the following fabrication process. (f) Calculated optical losses of the TE_0_ and TE_1_ modes in the TE_1_-mode filter. The inset shows the calculated modal ER of the TE_1_-mode filter with different widths of the PtSe_2_ nanoribbons. (g) Calculated optical losses of the TE_0_ and TE_2_ modes in the TE_2_-mode filter. The inset shows the calculated modal ERs of the TE_2_-mode filters with different widths of the PtSe_2_ nanoribbons.

Figure 1(a) shows schematics of the on-chip spatial mode filters. Asymmetric directional couplers can be used for energy conversion between the fundamental and high-order modes. Detailed designs of the asymmetric directional couplers can be found in the Appendices. Meanwhile, PtSe_2_-on-silicon waveguide devices were designed to develop TE_1_ and TE_2_-mode filters. As shown in Figure 1(b) and (c), the filters consist of ten-layer thick PtSe_2_ nanoribbons and multi-mode silicon waveguides that were designed on an SOI wafer with a 70-nm thick top silicon layer and a 2-μm thick buried oxide. We then theoretically optimized the proposed spatial mode filters by using finite-element method software (COMSL Multiphysics). Figure 1(d) shows the effective refractive indices (RIs) as a function of the silicon waveguide width (*W*_WG_) at a wavelength of 2,200 nm. As the *W*_WG_ increases, more eigenmodes could be supported by the silicon waveguide. It is well known that, when *W*_WG_ is fixed, the higher the mode order, the higher the optical loss. On the other hand, when the mode order is fixed, the wider the waveguide, the lower the optical loss. Here, we chose *W*_WG−1_ = 3 μm for supporting the TE_0_ and TE_1_ modes and *W*_WG−2_ = 4.2 μm for supporting the TE_0_ and TE_2_ modes as a trade-off. Normalized electric-field intensity profiles across the silicon waveguide and simulated electric-field distributions of the TE_0_, TE_1_, and TE_2_ modes are presented in Figure 1(e). As for the TE_1_-mode filter, one PtSe_2_ nanoribbon was designed to be placed on the surface center of the TE_1_ mode waveguide, providing a large overlap with the electric field of the TE_0_ mode. Therefore, the energy of the TE_0_ mode can be filtered from the device. As for the TE_2_-mode filter, two PtSe_2_ nanoribbons were designed to be placed ±0.77 μm from the center of the TE_2_-mode waveguide surface, resulting in a significant absorption to the TE_0_ mode. Figure 1(f) and (g) present simulated optical losses of different modes in the TE_1_-mode and TE_2_-mode filters as a function of the PtSe_2_ nanoribbon width (*W*), where the parameters of PtSe_2_ used in the theoretical modal are obtained by fitting [38]. With the *W*_1_ and *W*_2_ increase, the optical losses of the waveguide modes increase. Especially the TE_0_ mode shows the most significant increase in optical loss, rising from 0.00337 dB/μm and 0.00273 dB/μm to 0.16526 dB/μm and 0.13939 dB/μm in TE_1_-mode and TE_2_-mode filters. Meanwhile, the optical losses of TE_1_ and TE_2_ modes vary moderately. We used the modal ER, defined as the ratio of transmittance of the pass mode and the filtered mode, as a figure of merit (FOM) of the proposed device. When there is no PtSe_2_ nanoribbon on the waveguide, the optical losses only depend on their propagation losses in the silicon waveguide. According to simulations, the TE_0_-to-TE_1_ and TE_0_-to-TE_2_ modal ERs without the PtSe_2_ can be calculated as 0.00988 dB/μm and 0.01796 dB/μm at 2,200 nm wavelengths. Theoretical results also show that when *W*_1_ = 1,200 nm and *W*_2_ = 1,500 nm, we can obtain a maximum TE_1_-to-TE_0_ modal ER of 0.07438 dB/μm and a maximum TE_2_-to-TE_0_ modal ER of 0.05659 dB/μm, as indicated in the insets of Figure 1(f) and (g). It is worthwhile to note that the ultrathin silicon waveguide with the deep subwavelength thickness not only increases the optical absorption which helps reduce the device footprints but also improves the fabrication tolerance due to low-index waveguide devices, benefitting MDM applications based on the PtSe_2_-on-silicon waveguide devices at 2-μm wavelengths and beyond, i.e. 3-μm wavelengths by using a suspended membrane waveguide [11].

## 3 Device fabrication and characterization

**Figure 2:**
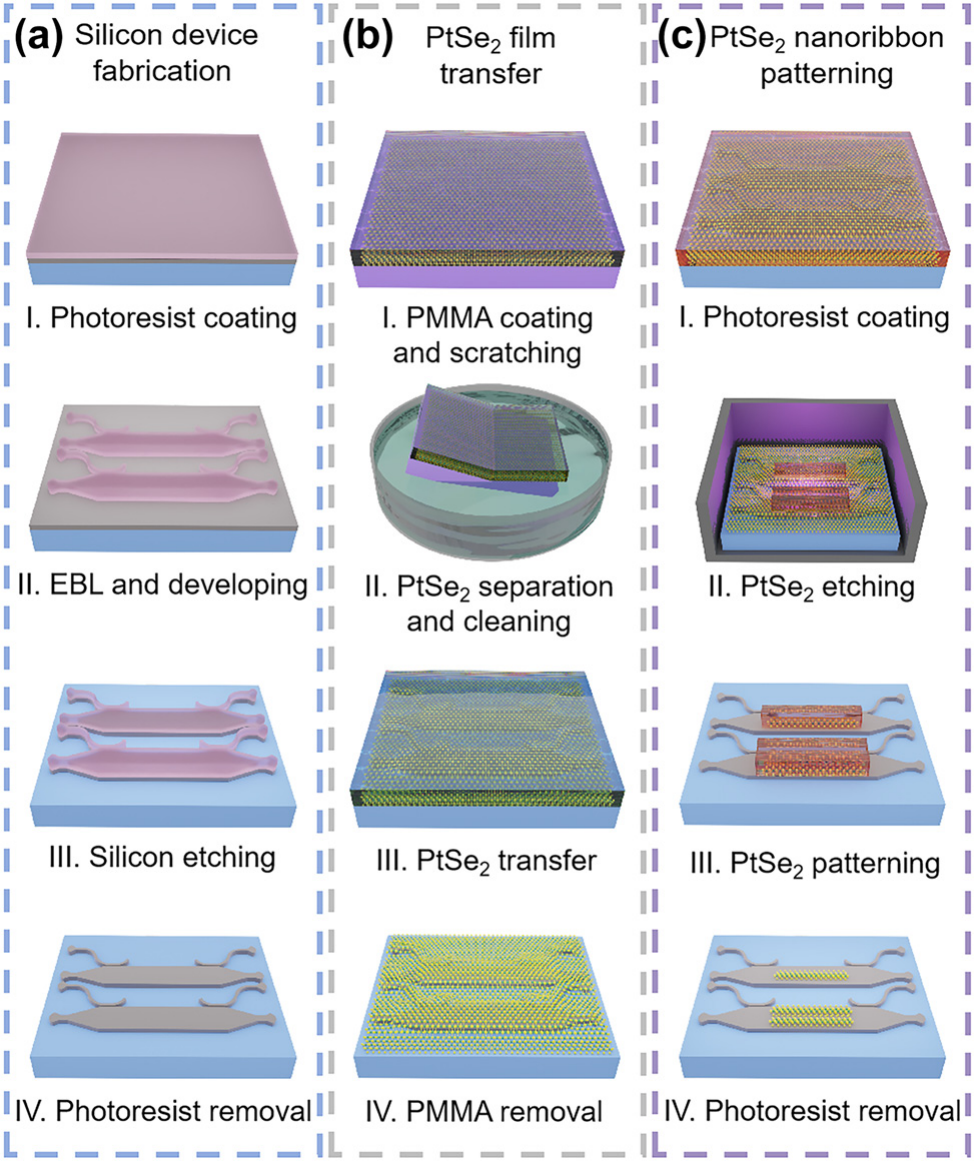
Fabrication of the proposed spatial mode filters. (a) Silicon waveguide device fabrication processes. (b) PtSe_2_ film wet transferring processes. (c) PtSe_2_ nanoribbon patterning processes.

Based on the theoretical analysis, we fabricated the designed spatial mode filters. The silicon waveguide devices were first fabricated by using electron-beam lithography (EBL) and reactive-ion etching (RIE) processes, as shown in Figure 2(a). In the second step, we transferred a PtSe_2_ film onto the fabricated silicon chip by using a modified wet transferring recipe, as shown in Figure 2(b). As for the recipe, a polymethyl methacrylate (PMMA) layer was spinning coated on top of a ten-layer chemical vapor deposition (CVD)-growth PtSe_2_ film on a silica substrate (SixCarbon Technology). Then, the PtSe_2_ film was separated from the silica substrate with the help of hydrofluoric acid wet etching and cleaned in deionized (DI) water. After that, the PtSe_2_ film was transferred onto the fabricated silicon chip. The PMMA was removed using an acetone solution, followed by cleaning it with anhydrous ethanol and DI water. Finally, the proposed devices were developed with the PtSe_2_ film patterning by using photolithography and argon plasma etching processes, as shown in Figure 2(c). More details of the PtSe_2_-on-silicon waveguide fabrication can be found in our previous study [38].

**Figure 3:**
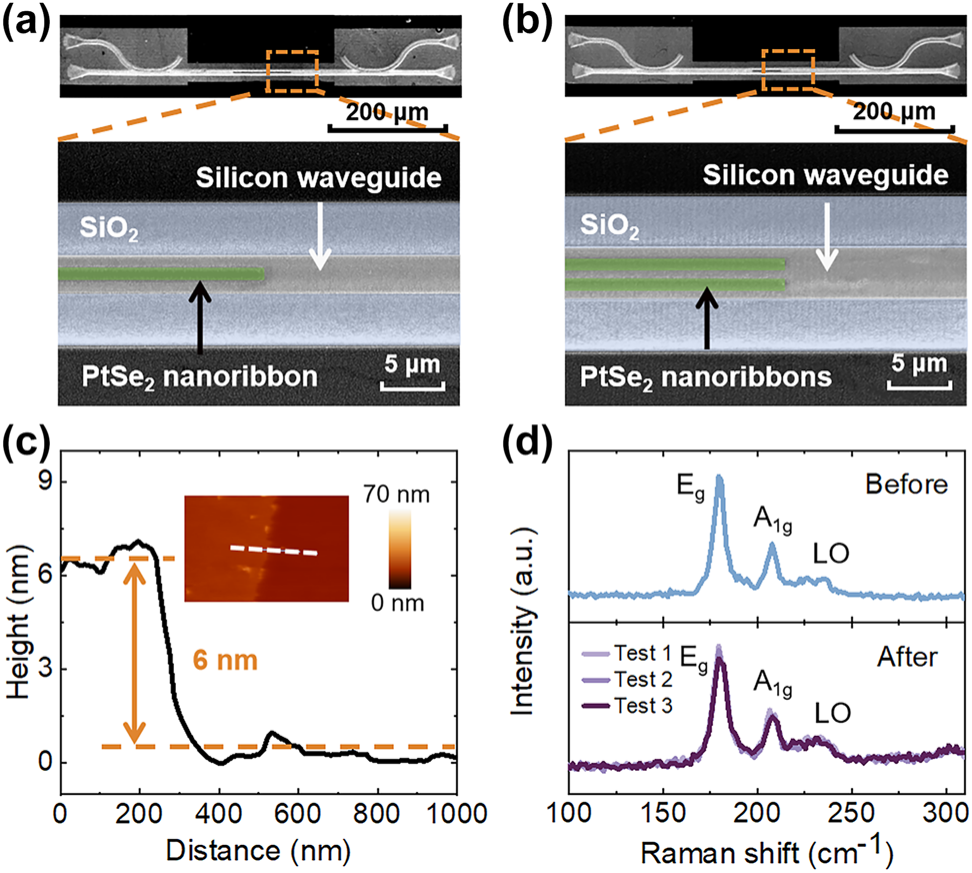
Characterization of the proposed spatial mode filters. (a) SEM image of the TE_1_-mode filter. (b) SEM image of TE_2_-mode filter. (c) AFM measurement of the PtSe_2_ film. The inset shows the height distribution of the PtSe_2_ film surface. (d) Raman spectra of the PtSe_2_ film before and after its transfer.

**Figure A.3:**
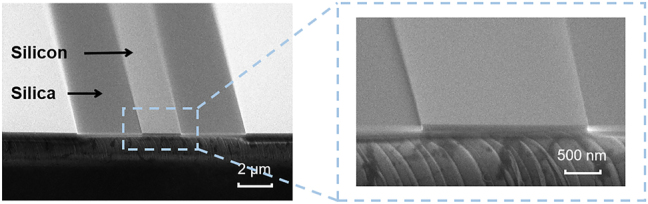
SEM images of the ultrathin silicon waveguide cross-section.

We characterized the fabricated spatial mode filters by using scanning electron microscopy (SEM), atomic force microscopy (AFM), and Raman spectroscopy. Figure 3(a) and (b) illustrate the SEM images of the TE_1_ and TE_2_-mode filters. We utilized grating couplers to couple the TE_0_-mode light into and out of the ultrathin silicon waveguides. 50-μm-long tapers on both sides of the multi-mode waveguide were used to adiabatically connect the multi-mode waveguide and single-mode waveguide without changing the mode order. The insets are the zoom-in images of the spatial mode filters. The cross-section view of the silicon waveguide is shown in Figure A.3. Moreover, the AFM measurement shows that the thickness of the ten-layer PtSe_2_ nanoribbon was ∼6 nm, as shown in Figure 3(c). In addition, the quality of the PtSe_2_ film was characterized by measuring the Raman spectra of the PtSe_2_ film before and after its transfer, as shown in Figure 3(d). Three characteristic peaks can be observed around 179 cm^−1^, 208 cm^−1^, and 235 cm^−1^, representing the E_g_ peak, A_1g_ peak, and longitudinal optical (LO) peak, respectively. The E_g_ peak has moderate variation, while three measurements from different positions show similar profiles, revealing the excellent quality of the PtSe_2_ film with good homogeneity and few defects after the wet transferring process [39].

## 4 Measurement and discussion

**Figure 4:**
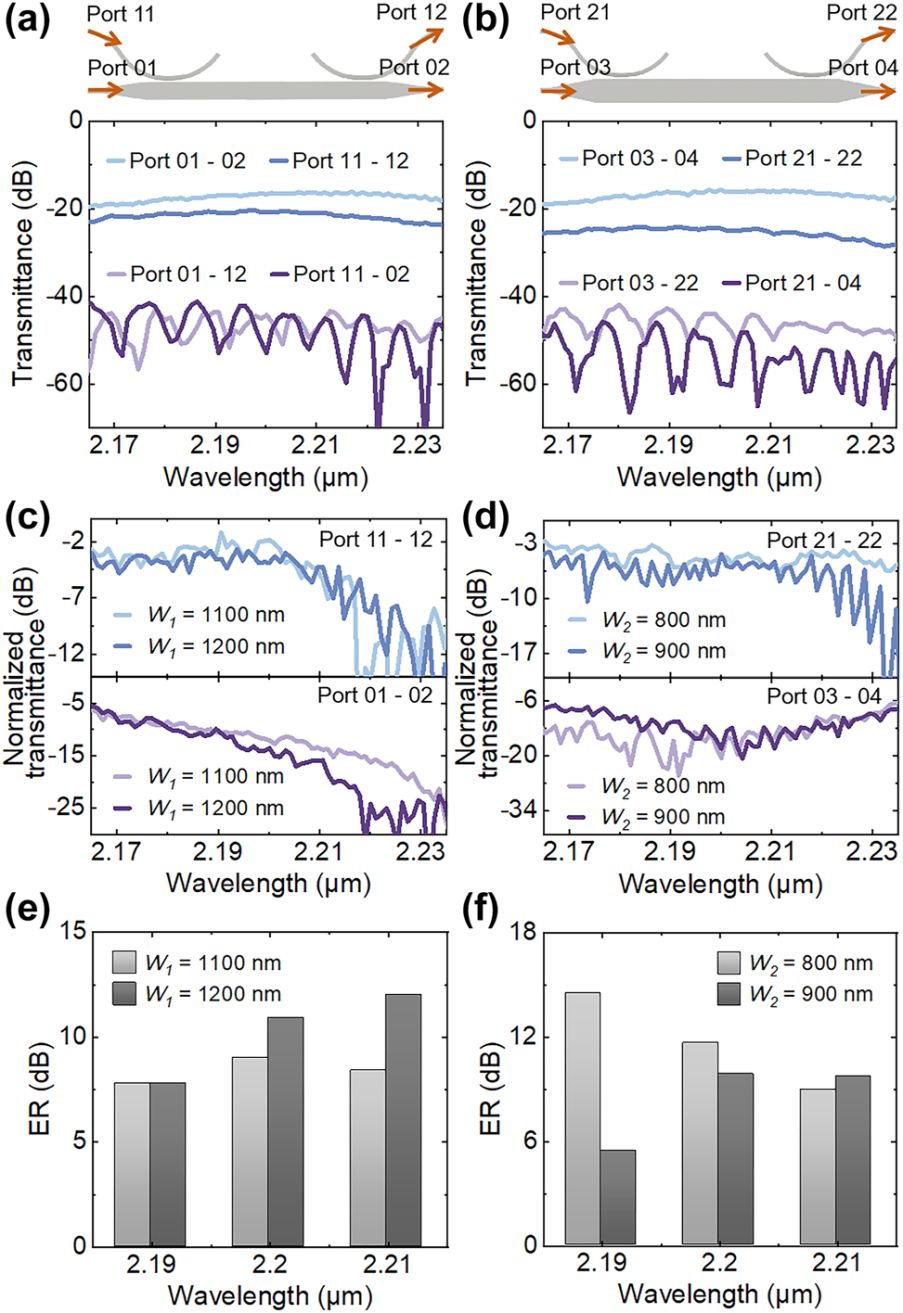
Experimental results of the fabricated spatial mode filters. Transmittance of the (a) TE_1_ and (b) TE_2_-mode filters before integrating the PtSe_2_ nanoribbons process. Transmittance of the (c) TE_1_ and (d) TE_2_-mode filters after the PtSe_2_ transfer and patterning. Measured modal ERs of the (e) TE_1_-mode and (f) TE_2_-mode filters.

Finally, we verified the experimental performance of the spatial mode filters. Figure 4(a) shows the transmission spectra of the silicon devices before integrating the PtSe_2_ nanoribbons process. When the light travels from Port 01 to Port 02 and from Port 11 to Port 12, the transmittance is much higher than that from Port 01 to Port 12 or Port 11 to Port 02, where optical losses are more than 45 dB. The TE_2_-mode filter without PtSe_2_ nanoribbons is also consistent with the description above, as shown in Figure 4(b). Here, due to the compact device footprints, the optical losses of the waveguide and taper can be neglected. The maximum coupling efficiency of the grating coupler was measured as −8 dB at the center wavelength of 2,200 nm, while the coupling losses of the TE_1_-to-TE_0_ and TE_2_-to-TE_0_ asymmetric directional couplers were ∼2 dB and ∼4 dB. After integrating the PtSe_2_ nanoribbons on the waveguides, the measured normalized transmittance around 2,200 nm wavelengths of the TE_1_ and TE_2_-mode filters, which are deducted the noises to avoid the interferences, are shown in Figure 4(c) and (d). Figure 4(e) and (f) display the modal ERs of the TE_1_ and TE_2_-mode filters. Here, considering the fabrication process and simulated results, we obtained four filters with the sizes of the PtSe_2_ nanoribbons set as *W*_1_ = 1,100 nm and 1,200 nm, *L*_1_ = 100 μm for the TE_1_-mode filters, and *W*_2_ = 800 nm and 900 nm, *L*_2_ = 75 μm for the TE_2_-mode filters. Due to the significant overlap of the PtSe_2_ nanoribbons with the evanescent fields of the ultrathin waveguides, the optical losses of the TE_0_ mode are more than 15 dB in the TE_1_ and TE_2_-mode filters. Besides, benefiting from the subtly designed sizes and positions of the PtSe_2_ nanoribbons, the maximum modal ERs are up to 12 dB for the TE_1_-mode filter and 14.5 dB for the TE_2_-mode filter. It can also be seen that there is less variation in optical losses as the width increases, which agrees well with the simulation. The results indicate that the spatial mode filters have the advantages of high filtering efficiency, high selectivity, generous tolerances, and low limitations in the fabrication process, being suited for use in on-chip photonic integration systems.

## 5 Conclusions

In summary, we demonstrated the on-chip spatial mode filters with PtSe_2_ nanoribbons to eliminate modal crosstalk at 2-μm wavelengths. Ten-layer PtSe_2_ nanoribbons were subtly designed and fabricated on 70-nm thick silicon waveguide devices to provide selective giant absorption to undesirable optical modes in silicon waveguides. Our experimental results show that the TE_1_-to-TE_0_ and TE_2_-to-TE_0_ modal ERs of more than 12 dB and 14.5 dB can be achieved in the PtSe_2_-on-silicon waveguides at 2200-nm wavelengths. Our study is expected to open an avenue toward developing high-performance spatial mode filters with optoelectronic materials with suitable bandgaps for on-chip MDM applications.

## Figures and Tables

**Figure A.1:**
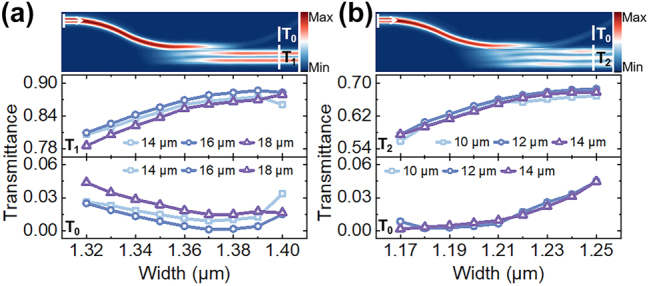
Design of the asymmetric directional couplers for mode conversion. Simulated transmittance of the (a) TE_1_-to-TE_0_ and (b) TE_2_-to-TE_0_ asymmetric directional couplers.

**Figure A.2:**
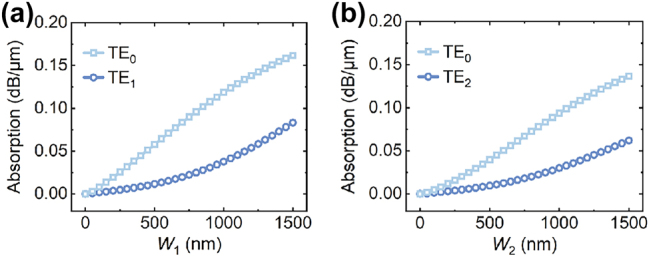
Simulated optical absorptions of the PtSe_2_ for different modes in the (a) TE_1_-mode filter and (b) TE_2_-mode filter.

## Appendix 1

We designed the asymmetric directional couplers for the mode conversion. As shown in Figure A.1(a) and (b), we optimized the coupling efficiencies of the TE_1_-to-TE_0_ and TE_2_-to-TE_0_ couplers by using a three-dimensional finite-difference time-domain simulator (Lumerical FDTD). The gap between the single-mode coupling waveguides and multi-mode waveguides was fixed at 150 nm. When the coupling waveguide lengths of the TE_0_-to-TE_1_ coupler and TE_0_-to-TE_2_ coupler were set as 16 μm and 12 μm, the transmittances of the TE_1_-mode (T_1_) and TE_2_-mode (T_2_) waveguides reached the maximum. We chose 1.37 μm for the TE_1_-to-TE_0_ coupler and 1.21 μm for the TE_2_-to-TE_0_ coupler as the single-mode waveguide widths, which agreed well with the phase matching conditions in Figure 1(d). The electric-field distributions with the above parameters show that the light could be well coupled from the single-mode waveguides into the multi-mode waveguides and excites the TE_1_ and TE_2_ modes.

## Appendix 2

With the *W*_1_ and *W*_2_ increase, the optical losses of the waveguide modes increase due to the increasing optical absorption of PtSe_2_. We simulated the optical absorptions within the PtSe_2_ for different modes, which have the same trends as Figure 1(f) and (g), as shown in Figure A.2.
